# Postgraduate digital health training programmes for primary care physicians: a scoping review protocol

**DOI:** 10.1136/bmjopen-2025-109715

**Published:** 2026-01-22

**Authors:** Sandra León-Herrera, Vinicius Anjos De Almeida, Sıdıka Ece Yokuş, Edmond Li, Sandro Rogerio Rodrigues Batista, Joana Teixeira, Ana Luisa Neves, Raquel Gómez Bravo

**Affiliations:** 1 Department of Psychology and Sociology, Universidad de Zaragoza, Zaragoza, Spain; 2 University of São Paulo, São Paulo, Brazil; 3 Department of Family Medicine, Faculty of Medicine, Manisa Celal Bayar University, Manisa, Turkey; 4 Department of Primary Care and Public Health, Imperial College London, London, England, UK; 5 Faculty of Medicine, Federal University of Goias, Goiânia, Brazil; 6 CINTESIS@RISE, Faculty of Medicine of the University of Porto, Porto, Portugal; 7 Rehaklinik, Centre Hospitalier Neuro-Psychiatrique (CHNP), Ettelbruck, Luxembourg; 8 Research Group Self-Regulation and Health, Institute for Health and Behaviour, Department of Behavioural and Cognitive Sciences, Faculty of Humanities, Education, and Social Sciences, University of Luxembourg, Luxembourg, Luxembourg

**Keywords:** Primary Care, Health informatics, Telemedicine, MEDICAL EDUCATION & TRAINING, Health Education, General Practice

## Abstract

**Abstract:**

**Introduction:**

The digital transformation of healthcare has created an urgent need for primary care physicians (PCPs) to acquire competencies in digital health. However, the structure and scope of postgraduate training programmes remain poorly defined and unevenly implemented worldwide, and no scoping review has yet synthesised the evidence. This review aims to map existing postgraduate digital health training programmes for PCPs, including their content, structure and delivery approaches.

**Methods and analysis:**

This scoping review will follow the Joanna Briggs Institute methodology and adhere to the Preferred Reporting Items for Systematic Reviews and Meta-Analyses extension for Scoping Reviews checklist. A systematic search will be conducted across five databases (PubMed, Scopus, Cochrane Library, ScienceDirect and Web of Science) and relevant grey literature, covering publications from January 2019 to June 2025. Studies describing postgraduate digital health training programmes for PCPs will be eligible for inclusion. Data will be extracted and synthesised descriptively and thematically using an inductive approach.

**Ethics and dissemination:**

As this study is based on a review of publicly available literature, ethical approval is not required. The findings will be disseminated through a peer-reviewed publication and conference presentations and will inform future curriculum development and policy in digital health education for PCPs. The results may also inform national curriculum reforms and accreditation standards, supporting more consistent and competency-based digital health education globally.

**PROSPERO registration details:**

This scoping review protocol has been registered with the Open Science Framework.

**STRENGTHS AND LIMITATIONS OF THIS STUDY**

- The review follows established Joanna Briggs Institute methodology and Preferred Reporting Items for Systematic Reviews and Meta-Analyses extension for Scoping Reviews reporting guidance for scoping reviews.
- A comprehensive search strategy, including multiple bibliographic databases and grey literature sources, is used to enhance coverage.
- Study selection and data extraction are conducted independently by two reviewers to improve methodological reliability.
- No formal quality appraisal of included sources is undertaken, consistent with scoping review methodology.
- The restriction to publications in English, French, Portuguese, Turkish and Spanish may result in the exclusion of relevant studies published in other languages.

## Introduction

 The rapid integration of digital health technologies into healthcare systems globally has led to a profound transformation in the delivery of medical services, particularly in primary care.^1 2^ Digital health encompasses a broad range of tools, including electronic health records (EHRs), telemedicine, mobile health (mHealth), artificial intelligence (AI) applications, remote monitoring, decision support systems and digital therapeutics.^3^,^5^ These innovations promise to enhance accessibility, efficiency, safety and personalisation of care.^5 6^ However, they also bring potential risks such as increased clinician workload, digital exclusion among vulnerable populations and concerns over data privacy.^7 8^

Primary care physicians (PCPs) are uniquely positioned to lead the adoption of digital health tools, given their central role in managing chronic conditions, coordinating care and ensuring continuity.^9 10^ However, evidence suggests that many PCPs feel unprepared to use digital tools effectively.^11 12^ A recent global survey by the WHO found that over 60% of PCPs reported low confidence in using digital health tools in clinical care.^13^ Barriers such as limited digital literacy, insufficient institutional support, lack of standardisation in training and uncertainty around the ethical and legal implications of digital health are commonly reported.^14 15^ These limitations can not only compromise the potential benefits of digital health innovations but may also widen disparities and contribute to clinician burnout.^14 15^

Over the past decade, health professional education has begun to adapt to the digital age.^16^ Medical schools, postgraduate programmes and continuing professional development (CPD) initiatives have gradually incorporated digital health content into their curricula.^17 18^ Yet, this integration has been uneven. While some institutions have developed structured, competency-based curricula covering areas such as teleconsultation, health informatics and AI in diagnostics, others rely on ad hoc workshops or informal learning opportunities.^19^,^21^ There is currently no consensus on what constitutes core digital health competencies for PCPs, although partial efforts—such as the WHO’s Global Strategy on Digital Health 2020–2025,^13^ the UK’s Topol Review,^22^ and the European Commission’s eHealth Action Plan^23^—have begun to outline foundational skills and learning outcomes. However, no unified framework has yet been adopted in postgraduate primary care training.^24 25^

The importance of formal digital health education in postgraduate training has become more evident since the COVID-19 pandemic, which accelerated the adoption of virtual consultations and remote care modalities.^26^ This sudden shift exposed significant gaps in training and highlighted the need for sustainable, high-quality digital health education pathways at the postgraduate level.^27^ Addressing these gaps is critical not only for safe technology integration but also for promoting equity, workforce resilience and digital readiness across diverse healthcare systems.^28^ Despite increasing attention to the topic in academic and policy circles, a comprehensive overview of existing training programmes in digital health for PCPs is lacking.^29^ Existing literature reviews have either focused broadly on digital health education across all specialties or have emphasised undergraduate medical training, leaving a gap in understanding how postgraduate training is responding to digital transformation in primary care.^30 31^ For instance, Smith *et al*^32^ reviewed digital competencies in undergraduate medical education, while Patel *et al*^33^ analysed specialty-level postgraduate training, with limited focus on primary care.

Moreover, as digital health tools continue to evolve rapidly, it is essential to ensure that educational approaches remain aligned with the competencies needed for safe and effective use.^34^ Understanding how training programmes are structured, what topics are prioritised, how knowledge is assessed, and who is responsible for curriculum development is critical to inform future educational strategies and policy.^35^ Mapping this landscape can also help identify common challenges in implementation, such as resource constraints, faculty preparedness and technological barriers, as well as highlight innovative solutions that can be adapted in diverse settings.^36^

In this context, we propose to conduct a scoping review to systematically map the characteristics of postgraduate digital health training programmes for PCPs. Specifically, this review will aim to identify the range of digital health topics currently included in postgraduate training, the structure and format of these programmes, the instructional and assessment methods used, and the key actors involved in their development and delivery. The findings will contribute to a clearer understanding of the global state of digital health education in primary care postgraduate training and inform the development of more standardised, competency-based approaches in the future.

## Materials and methods

### Design

This study will follow the methodological framework for scoping reviews developed by the Joanna Briggs Institute (JBI).^37^ The protocol is reported in accordance with the Preferred Reporting Items for Systematic Reviews and Meta-Analyses extension for Scoping Reviews (PRISMA-ScR). Where applicable, relevant elements from the PRISMA-P checklist will be considered to ensure clarity and transparency in protocol reporting (online supplemental material).

This scoping review protocol has been registered in the Open Science Framework (OSF), and it is available under the DOI: 10.17605/OSF.IO/HDC3P to promote transparency and reproducibility.^38^

### Study timeline

The scoping review was conceptualised in June 2025. The protocol was developed between June and July 2025. The literature search and study selection are planned for September 2025, with data extraction and analysis to follow from November to December 2025. The final synthesis and manuscript preparation are expected to be completed by February 2026.

### Eligibility criteria

The eligibility criteria are based on the Population–Concept–Context framework recommended by the JBI:^37^

- Population: PCPs engaged in postgraduate education (residency training, early-career programmes or CPD). Although CPD introduces heterogeneity, its inclusion is justified by the fact that many PCPs acquire digital health competencies through non-formal learning later in practice, especially in regions where structured residency programmes are less prevalent.
- Concept: educational initiatives and training programmes (formal or informal) that include content related to digital health, such as telemedicine, EHRs, health informatics, mHealth, AI in healthcare or digital decision support systems.
- Context: postgraduate medical education settings across any country or region. Both academic and non-academic institutions will be included.

The inclusion criteria for this scoping review have been established to ensure that only relevant studies are considered. Specifically, studies are included if they meet the following conditions:

- Studies published between 1 January 2019 and 30 June 2025.
- Any type of primary study (quantitative, qualitative or mixed-methods), as well as systematic reviews, scoping reviews, or rapid reviews, programme descriptions, institutional reports and grey literature.
- Articles in English, French, Portuguese, Turkish or Spanish. These languages were selected based on the research team’s linguistic competencies and their representation in major global medical education systems. Native speakers will be involved in the screening and data extraction process to ensure accuracy.
- Studies describing digital health education targeting PCPs at the postgraduate level. Interprofessional training programmes will be included if they contain specific content or components directly addressing PCP training needs.
- Content addressing aspects of training structure, delivery or evaluation.

The exclusion criteria for this scoping review have been established to ensure that irrelevant or out-of-scope sources are omitted. Specifically, sources are excluded if they meet any of the following conditions:

- Studies focused exclusively on undergraduate medical education.
- Training not related to digital health.
- Editorials, commentaries, conference abstracts without full text, or protocols without results.
- Articles not available in English, French, Portuguese, Turkish or Spanish.

### Information sources

A comprehensive search will be conducted across five bibliographic databases to identify relevant literature. These databases include PubMed, Cochrane Library, ScienceDirect, Scopus and Web of Science. This selection ensures a broad coverage of biomedical, health sciences and interdisciplinary research relevant to digital health education.

In addition to these databases, grey literature sources will be systematically searched to capture unpublished, non-peer-reviewed or non-indexed materials that may provide valuable insights. The grey literature search will include OpenGrey, Google Scholar (limited to the first 200 results to maintain feasibility and minimise algorithmic relevance bias), ResearchGate, and websites of key professional and academic organisations such as the WHO, the World Organisation of Family Doctors (WONCA), the American Medical Informatics Association, and the Association of American Medical Colleges. Documents such as newsletters, policy briefs, white papers and reports will be included where relevant.

This multifaceted search strategy aims to ensure a thorough identification of relevant sources, reducing publication bias and increasing the comprehensiveness of the scoping review.

### Search strategy

The search strategy was developed through an iterative process by the research team, drawing on expertise in digital health and scoping review methodology. An initial exploratory search conducted in PubMed was used to identify relevant articles and to examine key terms used in titles, abstracts and indexing. These terms informed the development of a comprehensive search strategy combining controlled vocabulary (eg, MeSH terms) and free-text keywords related to digital health, postgraduate education and PCPs.

The finalised search strategy has been adapted to the syntax and indexing requirements of each database. Boolean operators (‘AND’, ‘OR’) will be used to structure searches appropriately across databases.

Table 1 presents the predefined search strategies for each database, including the key terms and filters to be applied. Searches will be limited to studies published between 1 January 2019 and 30 June 2025. Any deviations or refinements made during the execution of the searches will be documented and reported in the final scoping review to ensure transparency and reproducibility.

**Table 1:** Draft search strategies by database

Database	Search strategy
PubMed	(“digital health”(Title/Abstract) OR “eHealth”(Title/Abstract) OR “telehealth”(Title/Abstract) OR “telemedicine”(Title/Abstract) OR “health informatics”(Title/Abstract) OR “medical informatics”(Title/Abstract) OR “connected health”(Title/Abstract) OR “remote consultation”(Title/Abstract) OR “artificial intelligence”(Title/Abstract)) AND (“postgraduate education”(Title/Abstract) OR “residency training”(Title/Abstract) OR “medical education”(Title/Abstract) OR “postgraduate training”(Title/Abstract)) AND (“general practitioners”(Title/Abstract) OR “family physicians”(Title/Abstract) OR “family doctors”(Title/Abstract) OR “primary care physicians”(Title/Abstract) OR “GPs”(Title/Abstract)) AND (“2019/01/01”(Date - Publication): “2025/06/30”(Date - Publication))
Web Of Science	TS=(“digital health” OR “eHealth” OR “telehealth” OR “telemedicine” OR “health informatics” OR “medical informatics” OR “connected health” OR “remote consultation” OR “artificial intelligence”) AND TS=(“postgraduate education” OR “postgraduate training” OR “residency training” OR “medical education”) AND TS=(“general practitioners” OR “family doctors” OR “family physicians” OR “primary care physicians” OR “GPs”) AND PY=2019–2025
Scopus	TITLE-ABS-KEY(“digital health” OR eHealth OR telehealth OR telemedicine OR “health informatics” OR “medical informatics” OR “connected health” OR “remote consultation” OR “artificial intelligence”) AND TITLE-ABS-KEY(“postgraduate education” OR “postgraduate training” OR “residency training” OR “medical education”) AND TITLE-ABS-KEY(“general practitioners” OR “family doctors” OR “family physicians” OR “primary care physicians” OR GPs) AND PUBYEAR >2018 AND PUBYEAR <2026
ScienceDirect	(“digital health” OR eHealth OR telemedicine) AND (“postgraduate education” OR residency) AND (“general practitioners” OR “family doctors”)
Cochrane Library	(“digital health” OR eHealth OR telehealth OR telemedicine OR “health informatics” OR “medical informatics” OR “connected health” OR “remote consultation” OR “artificial intelligence”) AND (“postgraduate education” OR “postgraduate training” OR “residency training” OR “medical education” OR curriculum OR “training programme”) AND (“general practitioners” OR “family doctors” OR “family physicians” OR “primary care physicians” OR GPs)

### Study selection and data management

All retrieved records will be imported into Mendeley Reference Manager, where automatic deduplication will be performed. The study selection process will consist of two sequential screening phases:

- Title and abstract screening: two reviewers will independently screen the titles and abstracts of all identified records against the predefined inclusion criteria. Studies that clearly do not meet the criteria will be excluded at this stage. Any discrepancies between reviewers will be discussed and resolved by consensus.
- Full-text screening: articles deemed potentially eligible will be retrieved in full and independently assessed by the same two reviewers. Reasons for exclusion at the full-text stage will be recorded in detail to ensure transparency and reproducibility.

In cases where disagreements persist after discussion, a third reviewer will be consulted to reach a final decision.

Throughout the selection process, eligibility criteria will be applied consistently to minimise bias. Reviewers will pilot-test the screening criteria on a sample of records prior to full screening to ensure consistency in interpretation.

The study selection process will be documented using a PRISMA-ScR flow diagram, which will include the number of records identified, screened, assessed for eligibility and included in the final synthesis, along with reasons for exclusion at each stage.

### Data extraction

A standardised data extraction form will be developed specifically for this review and will be pilot tested to ensure consistency and clarity. Two reviewers will independently extract data from the included sources. Key variables will include bibliographic details, study design and characteristics of the postgraduate programme. Information will also be collected on the intended target audience, digital health topics addressed, curriculum structure and content, and the instructional formats used (table 2).

**Table 2:** Data extraction variables

Category	Variables to be extracted
Bibliographic information	Author(s), publication year, country
Study characteristics	Study design
Programme characteristics	Type of postgraduate programme, target audience (eg, residents, early-career PCPs)
Educational content	Digital health topics covered, curriculum structure and content
Instructional design	Format and delivery methods (eg, in-person, online, blended)
Evaluation	Assessment strategies or evaluation approaches used
Developers/implementers	Institutions or organisations responsible for development and implementation
Implementation factors	Reported challenges, barriers or facilitators

PCPs, primary care physicians.

Additionally, the extraction will capture any evaluation or assessment strategies applied within the programmes, details about the institutions or organisations responsible for developing and implementing the programmes, the use of pedagogical frameworks (eg, competency-based learning, adult learning theory), and any reported challenges or barriers related to their implementation. Any discrepancies in data extraction will be resolved through discussion and consensus between the two reviewers; if needed, a third reviewer will adjudicate unresolved disagreements.

The data extraction tool will be shared publicly via the OSF to promote transparency and reproducibility.

### Data synthesis

Extracted data will be synthesised using a descriptive and thematic approach, consistent with the objectives of a scoping review. Quantitative information will be summarised narratively, highlighting patterns, similarities and differences across sources without performing statistical analyses.

Qualitative data will be analysed thematically using an inductive approach, following the six-phase method of Braun and Clarke.^39^ This process will involve open coding to identify recurring concepts, which will then be grouped into broader themes through iterative categorisation. An iteratively developed codebook will guide the coding process, and the final version will be made available as online supplemental material to enhance transparency and reproducibility. Coding will be conducted using NVivo (or equivalent software).

To ensure reliability and consistency, at least two reviewers will independently code the data. Themes will be refined collaboratively through a consensus-building process that involves multiple coders and iterative rounds of discussion. If disagreements persist, they will be resolved with the involvement of a third reviewer. The final themes will reflect both common patterns and contextual variability across the included programmes.

Synthesised findings will be presented in summary tables, thematic maps, or visual displays (eg, matrices or conceptual diagrams) to describe training structures, content, delivery modes and implementation strategies. These outputs will aim to provide a comprehensive overview of current postgraduate training in digital health and identify potential gaps and areas for further development.

### Quality appraisal

Consistent with the objectives of scoping reviews, no formal risk of bias or methodological quality assessment will be conducted.^40^

### Ethics and dissemination

Ethical approval is not required for this review, as it will only include data from publicly available sources. The review will be conducted with rigour and transparency to ensure reliability and reproducibility.

For matters related to research integrity, the institutional contact is the Research Governance and Integrity Team, Imperial College London (email: rgitcoordinator@imperial.ac.uk).

The results of the review will be submitted for publication in a peer-reviewed, open-access journal and presented at academic conferences. Summary findings will be shared with relevant stakeholders in medical education and digital health policy. Online supplemental materials, including search strategies and data extraction templates, will be made publicly available via the OSF.

### Patient and public involvement

None. Patients or members of the public were not involved in the design, conduct, reporting or dissemination plans of this scoping review protocol.

## Discussion

The rapid digitalisation of healthcare delivery, especially in primary care, has created an urgent need to ensure that healthcare professionals are adequately trained to navigate, apply and critically evaluate digital health technologies.^24^ This scoping review seeks to provide a comprehensive map of postgraduate training programmes in digital health targeted at PCPs—a professional group pivotal in leading and scaling digital transformation at the community level. By identifying what is currently offered and where gaps exist, the review will also help inform national and institutional policies to guide investment in workforce development.

Despite increasing recognition of the importance of digital health, there remains no standardised or widely accepted framework for its integration into postgraduate medical training.^41^ Educational efforts in this area vary widely across institutions, countries and specialties, and there is limited synthesised evidence on what digital health topics are prioritised, how they are taught and how learning outcomes are assessed.^42^ This variability not only risks creating uneven competencies across practitioners but also has broader implications for patient safety, healthcare quality and digital health equity.^43^

One of the main strengths of this scoping review is its focus on a critical and timely issue: how we are preparing PCPs for a digital future. By systematically identifying and describing existing educational initiatives, this study will help uncover exemplary practices as well as gaps in current approaches. The inclusion of diverse study types, geographic regions and grey literature sources will enhance the comprehensiveness of our findings and ensure that both formal academic programmes and informal or emerging models of digital health training are considered.

Furthermore, this review will explore not only curricular content but also pedagogical formats and evaluation strategies, offering a multidimensional perspective on how digital health education is operationalised in real-world postgraduate settings. By identifying who develops and delivers these programmes—be it universities, professional bodies, government agencies, or private sector actors—we also aim to illuminate the institutional landscape shaping this evolving field. The authors found no scoping reviews addressing this topic at the time this protocol was written.

A key output of this review will be a thematic framework of existing training models, organised by core topics (eg, telemedicine, EHRs, AI), delivery modalities (eg, e-learning, workshops, integrated rotations) and evaluation methods (eg, formative assessment, reflective practice, credentialing). This framework may serve as a foundation for future consensus-building efforts or validation studies aimed at developing internationally recognised competency standards in digital health.

However, certain limitations must be acknowledged. First, as a scoping review, this study will not assess the methodological quality or effectiveness of individual programmes, nor will it perform meta-analytical comparisons. Second, although the review expands language inclusion to English, French, Portuguese, Turkish and Spanish, relevant training programmes published in other languages may still be excluded, potentially underrepresenting practices in countries where these languages are not widely spoken. Third, the anticipated heterogeneity of study designs and reporting styles may limit the depth of synthesis possible in some domains.

Despite these limitations, the proposed review is expected to offer valuable insights into how the global primary care workforce is being equipped—or not—for the digital transformation of healthcare. By identifying common challenges, such as lack of standardisation, resource limitations or resistance to change, we hope to inform more strategic and coordinated efforts in educational policy, curriculum development and faculty training. Ultimately, this work aims to support a digitally competent, patient-centred and resilient primary care system, grounded in interdisciplinary and cross-sector collaboration.

## Supplementary material

10.1136/bmjopen-2025-109715 online supplemental file 1
