# Supplementary material for: Postgraduate digital health training programmes for primary care physicians: a scoping review protocol
Source: BMJ Open. 2026 Jan 22;16(1):e109715. doi: 10.1136/bmjopen-2025-109715 (PMC12829378; doi:10.1136/bmjopen-2025-109715)
Supplement: Supplementary file 2 [file bmjopen-2025-109715.reviewer_comments.pdf]

PEER REVIEW HISTORY

BMJ Open publishes all reviews undertaken for accepted manuscripts. Reviewers are asked to complete a checklist review form and are provided with free text boxes to elaborate on their assessment. These free text comments are reproduced below.

ARTICLE DETAILS

Title (Provisional)

Postgraduate Digital Health Training Programs for Primary Care Physicians: A Scoping Review Protocol

Authors

León-Herrera, Sandra; Anjos De Almeida, Vinicius; Neves, Ana Luisa; Yokuş, Sıdıka Ece; Li, Edmond; Batista, Sandro Rogerio Rodrigues; Teixeira, Joana; Gómez Bravo, Raquel

---

VERSION 1 - REVIEW

**Reviewer**	**1**
**Name**	**Farahani, Azam**
**Affiliation**	**Shahid Beheshti University of Medical Sciences, Department of Pediatric Nursing, School of Nursing and Midwifery**
**Date**	**19-Oct-2025**
**COI**	**None**

After careful review of the article titled "Postgraduate Digital Health Training Programs for Primary Care Physicians: A Scoping Review Protocol", it appears that the chosen method - the Scoping Review Protocol - is not well aligned with the research question posed. The research question appears to require a more analytical or empirical approach to provide meaningful evidence about the design or effectiveness of postgraduate digital health training programs. As a result, this approach does not adequately address the research objective.

For these reasons, I consider this article unsuitable for publication in its current state and recommend its rejection.

**Reviewer**	**2**
**Name**	**Coventry, Jessica**
**Affiliation**	**Monash University**

**Date**	**02-Dec-2025**
**COI**	**None**

Thank you for the opportunity to review this scoping review protocol. The topic is interesting and I look forward to reading the review. Overall, the manuscript is clearly written and follows the recommendations for scoping review protocols. I have a few small recommendations which I believe will improve the transparency of this protocol.

Introduction

The introduction appropriately identifies the research gap. A few sentences referencing existing literature appear to be missing citations.

For example: page 3: "However, evidence suggests that many PCPs feel unprepared to utilize digital tools effectively" and "Barriers such as limited digital literacy, insufficient institutional support, lack of standardization in training, and uncertainty around the ethical and legal implications of digital health are commonly reported."

Method

Please cite JBI reference: "The eligibility criteria are based on the Population–Concept–Context (PCC) framework recommended by the JBI:"

In exclusion/inclusion criteria you have include 'review papers' but in exclude you have 'non-systematic reviews. Currently a scoping review or rapid review would sit as both include and exclude. Please revise.

Data synthesis

You have specified that qualitative data will be analysed thematically please state and cite what method you will be using (for example Braun and Clarke's thematic analysis).

In your last paragraph you state "to illustrate the diversity of training structures, content, delivery modes, and implementation strategies" while diversity is highly likely, this anticipates your results which should be avoided in a protocol. Please reword.

VERSION 1 - AUTHOR RESPONSE

Reviewer 1:

Requested changes	Changes made
**Reviewer**:<br><br>After careful review of the article titled "Postgraduate Digital Health Training Programs for Primary Care Physicians: A Scoping Review	**Authors**:<br><br>"We thank the reviewer for their careful assessment. We respectfully note that, as indicated by the Editor, this concern was not considered valid following

Protocol", it appears that the chosen method - the Scoping Review Protocol - is not well aligned with the research question posed. The research question appears to require a more analytical or empirical approach to provide meaningful evidence about the design or effectiveness of postgraduate digital health training programs. As a result, this approach does not adequately address the research objective.<br>For these reasons, I consider this article unsuitable for publication in its current state and recommend its rejection.	editorial evaluation and feedback from the second reviewer. Consequently, no changes were made in response to this comment, in line with the Editor's guidance.

Reviewer 2:

Requested changes	Changes made
**Reviewer**:<br><br>Thank you for the opportunity to review this scoping review protocol. The topic is interesting and I look forward to reading the review. Overall, the manuscript is clearly written and follows the recommendations for scoping review protocols. I have a few small recommendations which I believe will improve the transparency of this protocol.	
**Reviewer:**<br><br>"Introduction<br><br>The introduction appropriately identifies the research gap. A few sentences referencing existing literature appear to be missing citations.<br><br>For example: page 3: "However, evidence suggests that many PCPs feel unprepared to utilize digital tools effectively" and "Barriers such as limited digital literacy, insufficient institutional support, lack of standardization in training, and uncertainty around the ethical and legal implications of digital health are commonly reported."	
**Reviewer:**<br><br>"Method<br>Please cite JBI reference: "The eligibility criteria	

are based on the Population–Concept–Context (PCC) framework recommended by the JBI:"<br><br>In exclusion/inclusion criteria you have include 'review papers' but in exclude you have 'non-systematic reviews. Currently a scoping review or rapid review would sit as both include and exclude. Please revise."	
**Reviewer:**<br>"Data synthesis<br>You have specified that qualitative data will be analysed thematically please state and cite what method you will be using (for example Braun and Clarke's thematic analysis).<br><br>In your last paragraph you state "to illustrate the diversity of training structures, content, delivery modes, and implementation strategies" while diversity is highly likely, this anticipates your results which should be avoided in a protocol. Please reword."	

VERSION 2 - REVIEW

**Reviewer**	**2**
**Name**	**Coventry, Jessica**
**Affiliation**	**Monash University**
**Date**	**15-Dec-2025**
**COI**	

Thank you for the opportunity to review another iteration of this manuscript.

I believe the edits made have strengthened the quality of the protocol and I am happy to endorse its publication.

In future, please provide responses to reviewers for all reviewers.
